# Nursing Section

**Published:** 1904-01-30

**Authors:** 


### The Hospital.

### Nursing Section.

Contributions for this Section of "The Hospital" should be addressed to the Editor, "The Hospital" Nursing Section, 28 & 29 Southampton Street, Strand, London, W.C.

No. 905.—Vol. XXXV. *SATURDAY, JANUARY* 30, 1904.

### Notes on News from the Nursing World.

### THE NURSING STAFF AT OSBORNE HOUSE.

Last week we announced that Miss Haines had been appointed, and had taken up, her duties as matron of Osborne House, now, thanks to the generosity of the King, the Convalescent Home for officers of the Army and Navy. The matron, we understand, will be assisted by four sisters, three of whom will be on day duty, one for each floor, and the other on night duty. There is a dining-room for the exclusive use of the nursing staff, and every possible provision has been made for their convenience and comfort.

### THE NEW RULE OF THE PENSION FUND.

A very important new rule is announced by the managers of the Royal Nationa ; Pension Fund. Henceforth, policy-holders who withdraw from the Fund after their policies have been in force seven full years will pay no administrative cost, but will receive their total payment with 2½ per cent. compound interest. The advantage of this to nurses who join the Fund with the view of belonging to it for a certain number of years is obvious. But we think that the new rule may also, by encouraging the habit of saving, have the very desirable effect of inducing many to remain in the Fund longer than they first intended, if not until the pension becomes due. The primary object of the Fund is, of course, the promotion of thrift among nurses, in order that they may enjoy an income when they can no longer earn one, and the new rule can scarcely fail to contribute, in a material manner, to that result.

### A POOR-LAW MATRON ON STATIONARY SISTERS.

An interesting contribution to the question how frequently it is desirable in the interests of patients and nurses to change the sister of a ward, comes from the matron of St. Marylebone Infirmary, in an interview with our commissioner, which appears in another page. Miss Ramsden, who, by the way, proves conclusively that so far as St. Marylebone Infirmary is concerned, hospital-trained nurses do not get all the best appointments, does not think that sisters should be moved frequently. But both in the interests of the patient and of the sister, she is of opinion that a move should be made every third or fourth year. This, it will have been observed, is the view of "Hospital Sister," who in her letter last week protested against appointments which are "practically for life." The matron of St. Marylebone Infirmary adds that "A good deal depends upon the woman," and upon whether the work becomes monotonous. Whatever differences of opinion may prevail in respect to stationary sisters, we believe that everyone will agree with the conclusions of Miss Ramsden, that as soon as the work becomes monotonous it should be changed. A sister who finds her work monotonous is in imminent danger of doing it badly.

### CHURCH NURSES' GUILD AT WIMBORNE HOUSE.

On Thursday last week a large number of hospital and district nurses, members of the Church Nurses' Guild, availed themselves of the invitation of Lady Wimborne to spend an afternoon at Wimborne House, Arlington Street. The company also included several ladies and gentlemen, among whom were Lady Borwick, Mrs. Lockyer-Lampson, lady president of the Guild, the Hon. Mrs. Eliott, hon. sec., and Sir Henry Burdett, who gave an address explaining the objects and methods of work of the Royal National Pension Fund for Nurses. In the course of his remarks Sir Henry said that it was the most difficult thing in the world to induce women to save, but when once they were converted to the doctrine of thrift they were most earnest in their endeavours. Fourteen years ago, at the time the Fund was started, Miss Florence Nightingale stated that the salaries of nurses were so meagre that it was impossible to save money. Yet now, in 1904, the Fund was the wealthiest beneficent society supported by working women. A programme of music was performed during the afternoon, light refreshments were provided, and each nurse was presented with a useful gift before she left.

### EDINBURGH ROYAL INFIRMARY AND OUTSIDE PROBATIONERS.

The period of training for probationers at Edinburgh Royal Infirmary has been increased to three years in all cases. This means that the Scottish Branch of the Queen Victoria's Jubilee Institute who have hitherto sent 12 nurses to the infirmary to receive hospital training will now be excluded. Of course, the change is the subject of adverse criticism, but if the lady superintendent of the Royal Infirmary wishes to have only probationers who have been selected by herself undergoing training in the infirmary, and the Committee of Contributors approve, we do not see that there is any just cause for complaint.

### ONE TRAINED NURSE FOR TWELVE SICK WARDS.

We draw attention to our report in another page of the proceedings at the meeting of the St. Olave's Board of Guardians last week in reference to the letter of the medical officer of Ladywell Workhouse. Dr. Macnamara's communication will be read with more interest in the light of certain facts which he does not mention. There are 12 sick wards at Ladywell, three in each block, half for males and half for females. There is one nurse, not trained, in charge of each block with three attendants under her. At 12.30 these attendants go to dinner, and the block, containing 75 beds, is left entirely in control of the charge nurse. At this dinner the superintendent nurse is expected to preside. At 1.30 the charge nurses dine, and the superintendent nurse has her dinner with them. During that dinner there is not a single nurse in charge of the establishment, and the medical officer is practically out of the house. We believe that it has been suggested that one of the charge nurses should go, one from the male and one from the female side, to the first dinner, with half the attendants, the other half going to the second dinner, in which case the charge nurses could preside at the first mess, and leave the superintendent nurse free to attend on the medical officer. But, surely, there is a simpler remedy. Why cannot the matron, or assistant matron, preside at one of the dinners?

### DEATH OF A MISSION NURSE FROM PLAGUE.

We regret to hear of the death, from plague, of Miss Elizabeth Walden, a nurse in St. Catherine's Hospital, Cawnpore. It will be recollected that on October 3rd last an article on "Nursing in a Cawnpore Hospital, by an Old Queen's Nurse," appeared in our columns. The author was Miss Walden, who in that article related her own experiences since October 1900, when she first went out to India under the auspices of the Society for the Propagation of the Gospel in Foreign Parts. Her services were greatly valued by the Society, and we can understand that they are very much concerned to secure someone who will be able to take the place she has left vacant.

### GLASGOW MATERNITY HOSPITAL.

As already announced, the Central Midwives Board has recognised as an approved institution the Glasgow Maternity Hospital. The midwives trained at this institution ought certainly to be well able to cope with midwifery and to know their duties to the medical officer. Last year there were 1,325 patients in the hospital—700 mothers and 625 patients—as well as 2,132 district patients and 665 at the West End branch, where there is a midwife and two nurses. The number of maternity nurses trained was 105 of whom about 70 took the four months' course, including those for the different rural associations. There are many abnormal cases, and these with the Cæsarean sections and the number of serious operations render the training especially valuable. The directors of the hospital have converted a small ward into an operation-room, and the staff fully appreciate having everything at hand ready to sterilise for use, instead of having to prepare any ward at any hour for an operation. There are three resident doctors, two visiting physicians for the town, and five midwives in the hospital with seven district physicians to whom the nurses can always apply in extremity if they are far from the hospital. Miss J. A. Husband, the matron, has had eleven years' experience in two maternity hospitals.

### COMPULSORY ATTENDANCE AT CHURCH.

As we antipated in our issue of January 2nd the Local Government Board have signified to the Ecclesḥall Bierlow Guardians that attendance at Divine service at the workhouse chapel on Sunday mornings cannot reasonably be held to form part of the duties of the superintendent nurse. Miss Pascoe has therefore been officially cleared from the charge of insubordination, which we are of opinion should not have been brought against her. It is perfectly legitimate for a Board of Guardians, as it is for any other public or private body, to stipulate that a nurse entering their employment should attend service, and few nurses, we hope, would demur to the condition. But if no stipulation of the kind is laid down before the engagement of a nurse, she is under no legal obligation, and is guilty of no dereliction of duty in not attending.

### NURSES AND DANCING.

A dance for members of the nursing profession on a large scale during February is being organised at Glasgow. As the function is to be held in the Queen's Rooms, it is not open to the objection which applies to dancing in a building containing the sick and dying. But we do not see why there should be dances specially arranged for members of any particular profession. There is no reason why a nurse should not enjoy a dance, as well as a dinner, at the house of one of her friends at any time she is off duty. But she does not in that case go in her professional capacity. Why should she? No one thinks of organising a dance for the special benefit of governesses.

### THE REPORT OF THE DEPARTMENTAL COMMITTEE.

At a meeting of the Council of the Poor-law Unions' Association for England and Wales, which was held on Thursday last week, a deputation from the Council of the National Association of Masters and Matrons of Workhouses with regard to the report of the Departmental Committee on Nursing in Workhouses, was received. Mr. J. T. White, Master of Epsom Workhouse, said that the main object of the deputation was to ask the Council to adhere to its resolution on the nursing question as submitted to the annual meeting of the Association in November. The primary object in view was admittedly to induce the Association to use its influence to secure the maintenance of the Nursing Acts of 1897. We have no doubt that this object will be achieved, but it does not follow that therefore the President of the Local Government Board will ignore the recommendations of his own Committee. Strongly as we dissent from the proposal to create a qualified nurse with one year's training, there are many others that would be adopted with great advantage to nurses and patients alike. But it is really time that Mr. Long made up his mind, and unless he announces his views before Parliament meets, we hope that he will be asked why his decision is put off and when it may be expected.

### NORTHAMPTON AND COUNTY NURSING ASSOCIATION.

In sending us a report of the annual meeting of the Northampton and County Nursing Association Miss Lunn, the energetic superintendent, says: "Northampton is a most hardened place, so let us escape one of the gibes you so frequently fling at institutes running both private and district nursing staffs under one management." A gibe, as Miss Lunn knows, is "to cast reproaches and sneering expressions," and whatever may be our opinion as to the policy of combining two branches of nursing which are better kept apart, we have certainly not used sneering expressions in respect to it. There are many organisations which combine them and are doing excellent service to the community in spite of mistaken policy on a particular point. We freely concede that in the case of the Northampton Association the mistake seems to pay, as both its branches have a balance on the right side. But it is possible that Mrs. Hickson, who complained that her house-to-house collection for the district fund was last year the smallest she had yet realised, may have had refusals to contribute by persons who are under the impression that the receipts of the district branch are supplemented by the earnings of the private nurses. This, we are glad to say, is not so; the only contribution made by the private branch in the year being £7 4s., the remuneration of district nurses taking temporary duties on the private staff. The Northampton Association has been fortunate in securing Earl Spencer as the new president, and Miss Lunn and her capable nurses are to be congratulated upon the admirable record of work done both in town and county.

### SERIOUS CHARGES AGAINST A WORKHOUSE MATRON.

An inspector of the Local Government Board, Mr. Bircham, was occupied last week with an inquiry at Carmarthen Workhouse into serious charges made against the matron, and previously brought before the Guardians by a lady member of the Board. The evidence given at the inquiry was of a very contradictory character. Notably, a nurse who, according to the originator of the proceedings, said she had seen the matron drunk and unkind to inmates and sick patients, now greatly modified her statements, and declared that she had not witnessed any acts of cruelty. The fact that the inspector did not consider it necessary to swear the matron is significant, though the latter elected to be sworn, and having denied the truth of these particular charges, said that she had been 14 years under the supervision of Mr. Bircham and had never before been accused of intemperance or any other offence.

### THE ALLEGED BRAWLING AT HACKNEY INFIRMARY.

The Local Government Board have refused to institute an inquiry into the conduct of a Guardian of the Hackney Union accused by several of the nurses of brawling upon the occasion of his visit to the wards of the infirmary on Tuesday, December 6th. The reason given for this decision is that the Board "have no authority to adjudicate on the conduct of a Guardian." We regret that this should be the case, and we conclude that judgment in the matter will now be relegated to the ratepayers of Hackney, who, at the forthcoming election, will doubtless be concerned to ascertain whether or not the Guardian in question merits a renewal of their confidence.

### THE NURSE AND THE CURATE.

At Leeds, on Monday, Caroline Louie Fox, described as a certificated nurse, was summoned by the Rev. A. R. Light, curate of St. Michael's, Headingley, before the stipendiary magistrate, for alleged persistent annoyance extending over four years. The defendant, it was stated, was under the impression that Mr. Light was in love with her, and had been refused the Sacrament at the church by the vicar in consequence of her conduct. Continuing to molest the curate, she was compelled to sign a bond forfeiting £50 if she continued her course of persecution, but in less than a month afterwards she repeated the annoyance. In a letter to the Clerk of the Peace she wrote: "He will never marry another woman, never die without making peace with me, so the sooner he does it the better." Eventually, she was ordered to enter into a recognisance of £20 and to find one surety of £20 to be of good behaviour for 12 months. Miss Fox is a woman of 45.

### DISTRICT NURSES AND INSANITARY DWELLING-HOUSES.

The Duchess of Buccleuch presided at the annual meeting of the Dalkeith and District Nursing Association. The committee in their report deplored the terrible condition of many of the dwelling-houses visited by the nurse, who is leaving Dalkeith, and it may be hoped that her successor will be able next year to say that there has been an improvement. The district nurse who has the courage of her convictions in this respect, and tells her committee the truth, may give offence to a certain class of owners of property; but she is fulfilling a part, and not the least important part of her duty. Valuable as skilled nursing is to the sick poor, it is even better that sickness should be averted than that it should be alleviated; and district nurses who help, directly or indirectly, to reduce the number of insanitary dwellings are among the most useful of social crusaders.

### A QUESTION OF SERVIETTES.

The Plymouth Guardians have been occupied with the question whether the nurses in their employ should be provided with serviettes. It was observed that the master's estimate contained an item, "one dozen serviettes," and a Guardian inquired for whom they were wanted. The master stated that they were intended for the use of the nurses and would replace those which were worn out. The Guardian, protesting that "many ratepayers could not afford such luxuries," moved that the item be struck out, and found a seconder. The motion was, however, defeated, and the nurses are not therefore to be denied the use of serviettes at meals, which it may be added, are no more luxuries than tablecloths.

### SHORT ITEMS.

The sum of £1,000 has been contributed anonymously towards the erection of a nurses' home at Shottermill, Haslemere, and a valuable site has also been given.—An enjoyable entertainment was given to the in-patients of the Cancer Hospital last week by Miss Ethel Briscoe, of the Croydon Histrionic Society. The programme consisted of songs, etc., and the two sketches entitled "A Pair of Lunatics" and "Poor Pillicoddy."—During the past year 6,618 poor patients were nursed in their own homes by the staff of 99 nurses attached to the Plastow Maternity Charity and District Nurses' Home, and 2,241 persons were attended as out-patients. This is the largest total of any year in the charity's history.

### The Nursing Outlook.

"From magnanimity, all fear above';
From nobler recompense, above applause,
Which owes to man's short outlook all its charm."

### CLUBS FOR NURSES.

The Club for Nurses in Buckingham Street, Strand, held its annual meeting last Friday, and a report, or rather reports were adopted that call for comment. For it must be some ten or fifteen years since this club was first started in connection with the Midwives' Institute, and its progress, though slow, has been steady, until now it is a growing power in the nursing world. Last year there were over 7,000 visitors to the club and over 6,000 letters were sent out; many members got work through the registry; 760 books were taken out of the library, and several debates were held. Miss Amy Hughes, who is chairman of the debate committee, gave a very amusing account of the subjects dealt with and the experiences elicited; when an opener weighed the comparative merits of the old and the modern nurse, it was found that the modern nurse, if present, had nothing to say for herself, and all the praise was given to the training of ten years ago. These debates must be very instructive and teach more even than they amuse. On its more serious side the club reported four courses of lectures in preparation for the L.O.S. examination, and a series of post-graduate lectures to midwives by Dr. Mary Roche. Also its masseuse section told of two examinations at which 199 candidates presented themselves and only 38 failed. The Sectional Committee on Nursing issued a very vague report, also the District Centres Committee, but of course the Midwives branch had much to say. The demand for good midwives is great. On the whole it is an astonishing record of good quiet work, and it is to be hoped that the club, having achieved its main business point by the passage of the Midwives Act, will now develop along social lines and supply a good dressing-room and wholesome refreshments for its members. There is certainly room for such a club in London now that the prejudice against women's clubs has waned; but the difficulty will be always one of money. A nurse cannot afford a large subscription, and yet a place of rest and refreshment in the heart of London, a room where she can see the medical papers and the newest nursing books, is almost a necessity if she would keep up with the times. There is a reading and rest room in connection with the Royal British Nurses' Association, and there is a pleasant residential club in Mortimer Street. This latter is a great boon to private nurses and is on broad and comfortable lines; but in so far as it is proprietary and residential, it fails to meet the need of the country nurse who merely needs a *pied à terre* in London in order to learn the difficulties of government by being one of a responsible body.

Other professions for women have their clubs on a sound financial basis and on smooth running lines. Nor need the subscriptions be large for country members. True the Nurses' Club at present charges only 5s. a year to its midwife members, but this will have to be raised for newcomers should additions to the club premises be made. But there is a club for journalists which is exceedingly comfortable and of which the subscription for country members is only 10s. 6d., and for town members only a guinea a year. It is run with a membership of only some three or four hundred, and though its premises are small they are never crowded, because all who belong to it are busy women. We believe that some of the swell West End clubs which charge a ten-guinea entrance fee and ten-guinea subscription are often overcrowded simply because they are the resort of non-professional women whose time is not seriously occupied.

The life of a private or district nurse in a large town, if she live in some home or in her own lodgings, is often very isolated and dull. This is bad for the nurse and her work. It rubs the rust off the mind to meet one's comrades and compare experiences; it helps digestion to have conversation with one's meals, and the variety of food possible in a club is good for the general health. It is not only in London that this need is felt but also in such towns as Liverpool and Birmingham. If the club is not for nurses only, but for professional women as a whole, so much the better from the social side, but then the medical library and the nursing debates will probably be missing. Yet exactly as the Nurses' Club in Buckingham Street is broken up into detachments as it were—has its subcommittees of masseuses and so on—so a club for professional women in a provincial town could have its nursing and teaching sections, and the nurses could run their own meetings and manage their separate bookshelves.

The balance-sheet of the Nurses' Club is instructive as showing how businesslike are the arrangements and how wide the interests. The members' payments amount to only £184 out of the total receipts of £641; lecture fees, rents for letting the lecture-room and so on make up the rest. All sorts of societies hire the club's lecture-room for committee meetings regularly, and thus make the institution widely known and increase its power. It seems to us that the many interests which are represented and the excellent financial management augur well for the development of the club on the social side which is likely to come in the near future. It is not the least of the merits of the Nurses' Club that it is free from party politics and that at its tea-table the nurses of all hospitals and all institutions can alike enjoy the cup of peace.

### Lectures Upon the Nursing of Mental Diseases.

By Robert Jones, M.D.Lond., B.S., F.R.C.S.Eng., M.R.C.P.Lond., Resident Physician and Superintendent of the London County Asylum, Claybury.

### LECTURE IV.

The brain is the organ of the body quite as much as it is the organ of the mind. It is through the nervous system that man feels and moves, eats, breathes and excretes the waste material; grows, develops and propagates his kind. The activities of the body have to be provided for by taking in raw material in the form of food, and this food has to be reduced into simpler and more soluble substances before it can be taken into the blood. There is thus a continuous assimilation and destruction taking place in the human organism. This activity involves the production of heat, the regulation of the body temperature, and the liberation of energy which characterise life. This waste and repair is constantly going on, and the process by which living action is supported is termed *nutrition*. The conversion of raw substances into soluble and usable material proceeds through the aid of digestion. The products of digestion are taken into the blood and used by the cells of the body in the presence of oxygen, which is brought from the lungs into the lymph stream and thus placed in contact with the cells. The waste material is carried away by the blood to the excretory organs, such as the lungs, which give out carbonic acid, the skin which yields the perspiration, the kidneys which excrete or discharge the waste products of the urine, and the bowels which remove material unsuited in the office of nutrition. All these functions are under the control of the nervous system, and each or all of them may be affected in insanity. The term nutrition is thus applied:—(*a*) to the digestive processes which occur from the mouth to the alimentary canal; (*b*) to the taking up by the blood of these products of digestion; (*c*) to the distribution to the cells by means of the blood and lymph, of the nourishing material, and (*d*) to the building up of the cells of the living body into protoplasm, by means of these soluble materials. The nutrition of living tissues in man is effected through the *circulation of the blood.* A special term—*metabolism*—has been applied to define the complete series of changes which food undergoes to form protoplasm, and the decomposition of this protoplasm into more elementary waste products which are excreted.

*The Blood,* which forms 1-13th part of the weight of the body, is made up of solid *corpuscles*—white and red—floating in a fluid *plasma.* Blood is fluid in the living body, but when shed it clots into a jelly, the clot shrinks, so that when in a vessel the top part will be more fluid and transparent (serum) than the bottom (corpuscles and fibrin), for the corpuscles are heavier and fall down entangled in the fibrin. This fibrin has coagulated from the *fibrinogen*, which is fluid in the living blood, the coagulation being due to the breaking up of the white corpuscles. The *plasma* consists of water with salts and proteids (of which *fibrinogen* is one) in solution. When solidified, as occurs in drawn or shed blood, the liquid fibrinogen forms solid fibrin, and the clotting is due to this formation of fibrin. *Serum* is plasma without this fibrinogen. As stated, there are two kinds of corpuscles in the blood, the red, called *erythrocytes*, and the white, called *leucocytes.* There is only one white corpuscle to about 500 of the red. The red cells are essentially oxygen-carriers, and they carry oxygen owing to the presence in their structure of a complex fluid substance containing iron, called *hæmoglobin.* The oxygen is taken up by this substance to the lymph, which conveys it to the different cells in various parts of the body. When the red blood-corpuscles have parted with their oxygen, the hæmoglobin is thus *reduced* and the corpuscles must get fresh oxygen from the air of the lungs The heart and the brain have been proved to use a larger quantity of oxygen than any other organ, and they can only take up the oxygen through the presence of blood. The colour of the blood depends upon the amount of oxygen held by the hæmoglobin. When there is little oxygen, the colour of the blood is dark purple or venous; when fully charged with oxygen the colour is bright red or arterial. The red blood-corpuscles are formed within the marrow of spongy (cancellous) bone. They are constantly being destroyed after circulating for a time, and the red material in them is then changed into pigment which colours the bile, urine, etc. The red blood-corpuscles die in the spleen and the liver. The white blood-corpuscles or leucocytes are somewhat larger than the red. They are capable of independent movement, and they multiply in the blood by the division of each white cell into others, this being their usual mode of growth. They arise in the lymphatic glands, and are the scavengers of the body. They can assume all kinds of shapes, and can slip through the walls of the smallest capillary blood-vessels into the lymph spaces and into the tissues. They eat up dead tissue in a wound, which is the way that the slough of a wound or bed-sore is separated from the living tissues. They clear off the dust and smoke breathed into the lungs of town dwellers, carrying these sooty particles out of the air cells into the next lymphatic glands at the roots of the lungs, which are often seen to be quite black. They carry tattoo marks on the arms or hands into the glands of the armpit, and they also carry infection, such as that from the genital or other organs, into the glands of the groin, etc., which may break down and suppurate, leaving scars in consequence. It is through the white cells that bacteria are opposed when invading the body, for the white blood corpuscles devour the bacteria, and for this reason are occasionally called *phagocytes.* Leucocytes differ in size and in substance, and varying functions are allotted to the different kinds, of which about five varieties are usually described. The condition and the number of the blood corpuscles, both red and white, are important factors in regard to health. The sallow look of women suffering from the insanity of child-birth is probably due to blood changes induced by poisons absorbed into the blood. In cases of general paralysis of the insane, after congestive seizures, a definite nerve poison called cholin has been isolated and identified. The brittleness of the bones in the insane may also be due to blood changes brought about through disease of the nervous system. The conditions known as anæmia, leukæmia, and chlorosis need only to be mentioned as caused by abnormalities in the blood corpuscles.

The *lymph*, which is the fluid outside the capillaries, and which has really exuded from the blood-vessels into the tissues, forms a most important medium in the nourishment of the body. There are very few red corpuscles in the lymph, but many white, and a constant change goes on between the lymph in the lymph spaces and the blood in the capillaries. This change is very greatly influenced by the living cells which form the walls of the capillaries. If a poisonous insect sting the hand, the lymph is immediately poured out around the wound and the white corpuscles battle with the poison, carrying it to the nearest lymphatic gland, which becomes enlarged and tender. If the poison passes through the gland it then enters the veins and circulation, causing infection and a general disturbance. The lymph spaces surrounding the cells—which spaces are all connected into one great lymphatic system—end in fine tubes called lymph capillaries, and these unite into larger lymphatic vessels which follow the same course as the veins, having also valves like the veins. The lymphatics of both legs and abdomen collect together into a wide receiving vessel called the *receptaculum chyli*, which continues on into the angles of union of the veins of the left side of the neck and arm, whose lymph they also collect. The lymph of the alimentary canal is called *chyle* and it is conveyed by the *lacteals* to the thoracic duct. Anatomically the *lacteals* are not different from the lymphatics, although two absorbent systems are described. In the chest this vessel is called the *thoracic duct*. The lymph from the right side of the head and neck and upper part of the trunk is collected by the *right lymphatic duct*, and opens separately into the right vein of the neck. The lymphatic glands are situated in the course of the lymphatic capillaries which join freely with the spaces of the glands and into these spaces the white blood corpuscles enter, and by this means get into the general circulation. The quantity of lymph in the lymph spaces is greater than the amount of blood in the body, and it circulates mostly by the movements of the body and the contraction of the muscles. The permeability of the capillary wall and the rate of the diffusion of lymph depends upon many things, such as warmth, which increases molecular movement and favours what is termed *osmosis*—the flow from one side of a membrane to another; pressure, moisture, and the passage of an electric current also tend to help molecules to pass through a membrane. This is probably one of the reasons why electric baths and the general application of electricity is so beneficial in the insane, for it stimulates the nervous system to increased *metabolism*. The fact that the tissue cells are bathed by lymph, and are separated from the blood by the capillary wall, shows that in all interchanges between the blood and tissues the lymph must act as the medium of communication, and it has been demonstrated that the giving up of diffusible nourishment by the blood to the tissues and the taking up of the waste products through the intermediation of the lymph is carried out in the same way as are the gaseous interchanges, *i.e.*, by *diffusion*. The giving up of the proteid food from the blood to the tissues is different, for this does not diffuse, but oozes or transudes to the cells from the blood with the lymph. Marked disturbances of nutrition occur in the insane; there is great and rapid wasting, for instance, in acute mania and acute melancholia, whereas, in one stage of general paralysis of the insane a great increase in the weight of the body takes place.

### The Nurses of St. Marylebone Infirmary.

INTERVIEW WITH THE MATRON. BY OUR COMMISSIONER.

To St. Marylebone Infirmary belongs the distinction of being a pioneer institution in respect to Poor-law nursing reform. More than twenty years ago, when training nursing at workhouse infirmaries was quite the exception, the great building in North Kensington, so near and yet so far away from the centre of the Court suburb, was opened by the present King and Queen; while three years later Princess Christian formally inaugurated the school for probationers, and opened the first nursing home attached to a workhouse infirmary, which fitly bears the name of the Nightingale Home. There was a very special reason for this name being given to it, because, as Miss Grace Ramsden, the matron reminded me, after I had been over a considerable portion of the institution, the training school itself was started by St. Thomas's Hospital.

"How many probationers," I inquired, "formed the nucleus of the existing school?"

"Miss Vincent, my predecessor, the first matron, and Dr. Lunn came from St. Thomas's and commenced the training school with six probationers. Dr. Lunn, as you know, is still here, and is as energetic as ever. Miss Vincent remained for 19 years. The school was actually formed when the infirmary was opened, but for a time there was no home."

### The Affiliation to St. Thomas's.

"And you are still affiliated to St. Thomas's?"

"Yes, precisely as at the outset, except that the hospital now pay us the salaries of 15 probationers during their first year instead of six. Moreover, the Governors also help us in respect to the gratuity of £2 a year which we give to the probationers in their second and third years. This is a great help to us financially, and our association with such a hospital as St. Thomas's raises the standard. It is a unique position, and we appreciate it greatly."

"With the result, I presume, that you have as many candidates as you need for probationerships?"

"I am never short of them. In fact, I have the books full until the end of July. I think this shows that, so far as we are concerned, Poor-law nursing is not going backwards.'

### The Hospital-trained Nurses' Question.

"Were any of your staff nurses or sisters previously at St. Thomas's?"

"We have occasionally had both come here, and until lately our home sister has always come from St. Thomas's. When the last vacancy occurred, however, finding our own training so successful, we thought that we ought to provide our own nurses."

"Then it cannot be said of St. Marylebone Infirmary that hospital-trained nurses get all the best appointments?"

"It cannot, indeed. Not only was the home sister who was appointed two years ago, and the assistant matron who was appointed twelve months since, trained here, but so were all our sisters save one."

### The Nightingale Home.

"I suppose Miss Nightingale has always taken a great interest in the school?"

"She still maintains it. I go to see her frequently, and recently took her a photograph of a group of the probationers. She was very much pleased with it."

"I was particularly struck in going over the home by the fact that, though erected more than two decades ago, it will bear comparison with many modern buildings."

"Yes, I think it will; whether in respect to the accommodation or comforts provided. Each nurse has a bedroom to herself, and in addition to 51 bedrooms the home contains a sitting-room for the 'home sister,' a large sitting-room for the probationers, which is also used as a classroom, and a small sitting-room in which probationers can see their friends. The last is a great convenience."

"Are any meals served in the home?"

"Only afternoon tea, or anything light. The whole of the nurses dine in the infirmary, in the room in which you saw some of them at tea. The assistant-matron is in charge."

"Is there a library for the nurses?"

"A good library of medical and surgical books in the home, and a representative general library in the infirmary. There is a piano, but we have no gymnasium. I wish we

THE NURSES OF ST. MARYLEBONE INFIRMARY—*Continued.*

had one, for as I attach great importance to physical culture, I believe it would be of real service to the nurses."

### THE TRAINING SCHOOL.

"How many probationers are there now in the school?"

"Sixty-eight are undergoing training, but only 28 are in their first and second year. We call these probationers, and the remaining 40, who are in their third year, staff nurses. The other members of the staff are 12 day sisters, two night superintendents, and two assistant matrons."

"What is the period of trial?"

"Two months, and if at the end of that time candidates find that they are not strong enough or do not like the work, they go. But the majority remain with us for the three years."

"With prospects of becoming sisters?"

"Yes, as I mentioned there is only one sister who was not trained here, and she came from St. Thomas's 23 years ago, and is still most active. She has been in charge of the same surgical wards for 23 years."

### STATIONARY SISTERS.

"Then, she may be included among 'stationary sisters.' Are you in favour of the system of keeping a sister for a very long time in one ward?"

"No, as a rule I should not like sisters to remain in one ward for 23 years; but there are exceptions. I do not think that they should be moved frequently. But both in the interest of the patients and of the sister I should say that a move every third or fourth year is desirable, especially in Poor-law work. A good deal depends upon the woman. Also upon whether the work becomes monotonous, for when it becomes monotonous it begins to be irksome and should be changed. Our other surgical sisters take charge of the operating theatre for six months in turn."

"Do you get many operations?"

"Yes, a considerable number. Our new operating theatre which was only opened about a year ago is very completely appointed." Our probationers do student work, ten or twelve at each operation.

"It appears to come up to the standard of the best general hospitals."

"We think that it does, and also that the training here is equal to that of the best general hospitals. I was trained at the Royal Infirmary, Edinburgh, and can therefore say so more freely. Further, as I was afterwards sister for three years at the Liverpool Northern Hospital, and night superintendent at the Derbyshire Royal Infirmary, I can speak with experience of hospitals.

### COOKERY CLASSES.

"With regard to the theoretical training?"

"Lectures are given by Dr. Lunn, and the home sister has classes on physiology, anatomy, and massage. There is a bandage examination in the wards twice during the first year, and the Chairman of the Board of Guardians gives a prize. Cookery classes are also held."

"How many during the year?"

"Four in the quarter. The cook does the practical part, and makes beef tea, egg flip, blanc-mange, and other food for invalids; the home sister does the talking and superintends. These classes are very popular in the wards. There is a general examination at the end of the first year, which is always conducted by Dr. Sharkey of St. Thomas's Hospital. At the last examination all the probationers passed and several got the highest number of marks. Dr. Sharkey said that it was the most satisfactory examination he had held here."

### THE MATRON'S SIGNATURE.

"I conclude that you always sign the certificates which are given at the end of three years?"

"Of course I do, and I should not take a nurse here as sister whose certificate was not signed by the matron, or failing a matron by the superintendent nurse. The absence of the matron's signature takes Poor-law infirmary nursing off hospital lines. I regard the matron's testimony as of vital consequence. To dispense with it is bad alike for the nurses and for the training school."

### ON AND OFF DUTY.

"Before you tell me the hours on and off duty, will you kindly say how the nursing in the wards is arranged?"

"There are 744 beds, and each sister has charge of a flat with 62 beds. She has under her two staff nurses and one probationer during the day, and one staff nurse and one probationer in the night. All the probationers and staff nurses go on duty at 7 A.M., after breakfasting at 6.30. At 9 they have lunch, cocoa or hot milk, with bread and butter and jam. Part of them dine at 11.45, and the remainder at 12.15, all in the nurses' dining-room. They have tea at 4.30, and prayers are said in the board-room at 9, and all the nurses and domestic staff attend. We have nurses of all denominations including Roman Catholics."

"Are prayers said in the chapel?"

"No, because, unfortunately, as you observed, the chapel is upstairs and not easily accessible. After prayers, there is supper, bed at 10, and lights are out at 10.30. The night nurses breakfast at 7.45 P.M., go on duty at 8.30 P.M., and come off at 8.30 A.M. During the night they have meat or fish. Each member of the staff has an egg given her every day, and she can eat it when it is given to her, or eat it afterwards."

### THE DIET QUESTION.

"Have you any difficulty about diet?"

"None whatever. The diet is excellent, and so it ought to be. If an institution wants to keep good nurses, diet and everything that goes to make up a good home life must be considered."

"As to the time off duty," continued the matron, "the night nurses go out from 9 to 11 in the morning daily, and once a week from 9 to 1. They have a night off every month out of the five months they are on night duty. The probationers on day duty are off every day from 2 to 4 or 4 to 6, except one day, when they are only off for meals one evening from 4.30 to 9, and one afternoon."

"How about Sunday?"

"There is service in the chapel every Sunday morning, at which a third of the staff are always present. Those who are at the service are off duty in the afternoon; a third have the morning off; and the other third are off from 2 to 10 The sisters are off duty four afternoons from 2 to 4, one evening from 4 to 10, and one from 6 to 10. The staff nurses get almost the same time off duty as the sisters. With regard to holidays, the sisters have a month, the staff nurses three weeks, and at the end of the first year the probationers have three weeks."

### SALARIES AND RECREATION.

"What salaries do you give the probationers?"

"Ten pounds the first year, £20 the second, and £25 the third. The sisters start at £30, and rise to £32. Indoor uniform is provided, including a large cloak or shawl for each nurse to wear in passing across the bridges, or through the corridors. We have the open-air treatment in force both for surgical and for phthisical cases, and it is often very cold in the corridors. I also like nurses to wear goloshes when they go across the bridge in order to protect their feet from the damp."

"I imagine that you make the most of your admirable and extensive grounds."

"Yes, outdoor recreation is quite a feature here. Tennis and croquet are played, and every year we give a garden party, as well as the annual inspection at Christmas. Speaking of recreation, when a nurse is off duty for the day, she has a fire in her room, and can go in and out as she likes. I encourage the nurses to go to the theatre and to entertainments, because I am always afraid of the narrow institutional spirit being too strong."

NURSES' DINNER PARTIES.

"At Christmas, I think, there are special entertainments in the infirmaries for the nurses?"

"Yes, the sisters dine together one evening, and most of them afterwards go to the theatre, and have coffee when they return. The staff nurses do the same another evening, and while they are away the sisters and probationers work the wards. The probationers have their dinner also, to which they are allowed to ask friends, but they do not go out afterwards. Perhaps it may be of interest to add that the domestic servants also have a dinner of their own at Christmas, and one day in the week after Christmas 36 scrubbers and 19 washers sat down to roast beef and plum pudding."

"Do the nurses who are trained here do well in their post-graduate days?"

"They never experience any trouble in obtaining posts at home or abroad. Some belong to the Army Nursing Service Reserve, and others to the Indian Nursing Service. One of our nurses leaves for India next month, and another went about three months ago."

### The Superintendent Nurse at Ladywell Workhouse, and her Duties.

AT a meeting of the Board of Guardians of the St. Olave's Union on Thursday last week, under the chairmanship of Mr. W. H. Ecroyd, the following letter from Dr. J. T. Macnamara, Medical Officer of the Ladywell Workhouse was read:—

To the Clerk, St. Olave's Board of Guardians.

DEAR SIR,—I consider it my duty as medical officer of this workhouse to enter my emphatic protest against the decision arrived at by the committee on Friday last whereby the medical care of the sick poor is subordinated to purely domestic arrangements, and the most responsible officer in the house from a professional point of view is diverted from the specific duties for which she was engaged to undertake administrative work which legitimately she should not be called upon to perform.

The decision of the committee, if ratified by the board, practically debars the medical officer from entering the house during three of the most important hours of the day, and during these hours the superintendent nurse, who in his absence is responsible for the medical care of 800 old people averaging approximately 70 years of age, is called upon to perform duties entirely foreign to the object for which she was appointed. During these hours owing to difficulties in deglutition in people of that age and other causes accidents are particularly liable to happen; the sick wards are left with one charge nurse only in attendance for each block of 75 beds; or with attendants only to wait upon the sick. In face of the above statement, though I fear I cannot hope to alter the decision of the Committee, I must dissociate myself from any responsibility for any accident that may happen as a consequence of that decision.

The question being one which not only affects my professional position but also affects the general public and above all the residents of St. Olave's Union, especially those who now have or in the future may have friends or relatives inmates of Ladywell Workhouse, I take the liberty of asking you to read this communication to the Board, when the report comes up for consideration. I take the further liberty of stating that no arrangement which interferes with the free and unfettered performance of his duties by the medical officer, or which deprives him of the free and unfettered services of the superintendent nurse, can be other than detrimental to the best interest of the inmates.

Yours faithfully,
J. T. MACNAMARA, M.O.

P.S.—As the Committee's decision may not only prejudice my position as Medical Officer of Ladywell Workhouse, but may also be detrimental to my position as a private practitioner, I reserve to myself the right to publish this letter.

St. Olave's Workhouse, Ladywell, S.E.,
January 20th, 1904.

The recommendations of the Visiting Committee of the Ladywell Workhouse were to the following effect: That the consideration of the question of any permanent alteration of the duties of the superintendent nurse be adjourned for one month. That during that period the terms of the agreement of the Medical Officer as to his attendance be suspended, and he be granted the privilege of attending in the afternoon should he find himself unable to attend in the morning in time to complete the attendance upon his patients before 12.30 P.M. That the superintendent nurse be allowed to preside during the ensuing month at the nurses' and attendants' mess from 12.30 to 1.30, and that the Master and medical officer submit a report as to the working of these provisional arrangements at the end of the month.

Mr. D. McCarthy, Chairman of the Visiting Committee, stated that if the doctor visited the workhouse in proper time in the mornings the superintendent nurse would be at liberty to attend to all his cases, but the doctor alleged that he could not possibly find time to go round the workhouse in the morning, so as to allow the superintendent nurse to do as she had done hitherto. The committee had tried to meet the case by suspending for the time being the agreement that the doctor had entered upon to attend before 11 A.M., and permitting him to come in the afternoons. It appeared that the doctor wanted the superintendent nurse to be at his call at any time that he might go into the institution. He added that the Master said that matters would be much deranged unless he had the superintendent nurse to do a certain amount of administrative work in the house.

A long and discursive discussion took place.

The Rev. E. Scott Lidgett held that it was entirely beyond the powers of the committee, contrary to the rules of good management and highly inadvisable to lay down that the doctor was to do his work at certain hours. If the staff were insufficient to attend upon the doctor and do administrative work then it needed supplementing. If the doctor neglected his work or performed it irregularly, then it became a matter which the committee ought to be strong enough to hold him to. The help of the superintendent nurse should not be withdrawn from the doctor at whatever hours he came. It was quite impossible to say at what hours cases might not arise during the day, and then the doctor ought to have the services of the superintendent nurse or her representative. The Master's convenience ought to be subject to that of the doctor.

Another Guardian was of opinion that the doctor's letter was not such as should have been addressed to the Board by one of its officers, though he had a high opinion of the doctor's skill. He considered the suggestion that the doctor should attend before 11 A.M. absurd, and thought it wrong to interfere with the medical man, who should be the highest professional authority in the workhouse. There were 152 officers and servants, four charge nurses, and one superintendent qualified nurse, surely someone else could be found to look after the meals other than the superintendent nurse, whose duty it was to attend to the sick! He wished it to be understood that he was in no way taking the doctor's part for the language used in his letter, still he thought they should leave the methods of the institution in his hands and give him proper tools.

Mr. W. Shearring said that the doctor had practically consented to this arrangement. He considered that when the doctor was at the workhouse the superintendent nurse should be under his orders, but that he should suit himself to the requirements of the house. The Local Government Board would not give them any more superintendent nurses.

A resolution to the effect that the medical officer should be required to adhere to the terms of his agreement was put to the meeting and lost, as was also a further resolution that the matter should be referred to a general meeting of the whole Board the following week when the medical officer be requested to attend. The recommendations of the committee were then passed.

### Infirmary Nurse Burnt to Death.

GENERAL sorrow has been occasioned at Hackney Workhouse Infirmary by the sad death of Nurse Eliza Molyneux, through terrible injuries inflicted by burns. Our representative, who called on the matron, Miss Griffiths, was courteously supplied by her with full details of the catastrophe, which occurred on Wednesday evening last week. Nurse Molyneux was on night duty, and was seen by the receiving ward nurse to cross the quadrangle at twenty minutes to eleven, on her way to her cubicle. Ten minutes later, in reply to the assistant matron, who reminded her that it was getting late, Nurse Molyneux said she would be in bed in a minute. While brushing her hair by the fire, her flannelette night dress caught, and her long hair was quickly ignited. Attracted by her screams, Mrs. Barker, a scrubber, who was at work in an adjacent cubicle, rushed to the rescue, and threw some water from the toilet jug over her. The poor girl in her fright then ran into Nurse Mills's cubicle, still ablaze, and the latter, with great presence of mind, jumped out of bed and enveloped her in the bed-clothes. Another nurse, Miss Milton, with equal promptitude ran to the fire-hose, turned the water on to Nurse Molyneux and the creton curtain, which had caught the flames, and rang the alarm. The medical superintendent, Dr. Gordon, arrived almost immediately, followed by other officers, including the assistant matron and Miss Griffiths, who was under the impression that the fire drill was about to take place. Nurse Molyneux was promptly conveyed to a ward, where her burns, which were very severe, chiefly about the face, arms, and body, were dressed. Everything possible was done, but without avail, and at 11.35 the same evening she succumbed to her injuries. Her relatives were, of course, summoned, and one of her sisters was with her in a very short time, two others, who are nurses, arriving later. The Guardians, who met for their usual committee during the afternoon, made careful investigations, and it was found that nothing in the cubicle occupied by Nurse Molyneux was touched by fire. Her needlework—the bib of an apron—was as she put it down, with the needle in it, and a few books were lying on the bed, while in Nurse Mills's cubicle the only damage was a hole in the curtain. Nurse Molyneux was 23 years of age, and her parents reside in Birmingham. She entered the infirmary as a probationer on October 18th, 1901. Her work gave promise of high proficiency, and her disposition made her greatly beloved. She was previously at Poplar Infirmary.

At the inquest, held on Monday, the Coroner commented on the dangers of flannelette and the jury expressed sympathy with the relatives of the deceased nurse. The Clerk to the Guardians said that the patients would miss her very much. A verdict of accidental death was returned.

The Infirmary Committee has made a recommendation discouraging the use of flannelette and of celluloid hairpins, both being of a highly inflammable nature.

### Conference on the Requirements of the Midwives Act in Rural Districts.

ON Wednesday afternoon a conference to consider plans for meeting the requirements of the Midwives Act in rural districts was held under the auspices of the Rural Midwives Association at 3 Grosvenor Place by kind permission of Lady Esther Smith. There was a good attendance at the meeting, among those present being Lady Camoys, Mr. Heywood Johnstone, M.P., and Mrs. Heywood Johnstone, Sir Michael Foster, M.P., Dr. Percy Boulton, Dr. R. Boxall, Dr. W. S. A. Griffith, and Dr. C. Holman.

The chair was taken by Lord Belper, who said that, being an active member of the County Council's Association, and having taken interest in the Midwives Act, he was very glad to do anything towards making their proceedings of a businesslike character, and hoped they would have some satisfactory result. Their object in having this conference was by means of practical discussion to arrive at some general agreement with regard to the carrying out of the Act in rural districts. They were fully aware that the districts varied so much that any rules must necessarily be somewhat varied according to the circumstances of each case.

Mrs. Heywood Johnstone, Chairman of the Executive Committee, said that the work of the Association was to investigate the need for midwives in various localities, to give advice as to starting local associations, to train women for rural districts, and to give help as funds permitted to very poor parishes. Their one condition was that there should be a recognised scale of payment by the employer, and that the co-operation of the local doctor should be obtained. After mentioning the reduced fees to members, she went on to say that it was their desire to obtain sufficient funds to enable them to grant free training in certain cases. The Association was not yet a year old, but they had already received 99 applications, and had sent 23 persons to training institutions.

The discussion was then opened by Mrs. Heywood Johnstone, the first points taken being plans to meet the requirements of the Act in rural districts, and also the payment of midwives. Speaking individually, she said that two ways had been found of great assistance, adding midwives to the staff of local nursing associations and assisting local women to take up the work under the supervision of a small committee. It was thought best to pay the midwife a fixed salary, binding her for three years. The patients should be charged 10s. for the use of a midwife, 5s. to be returned if a doctor were not called in, and the doctor's fee to be paid by the Association.

Mrs. Rickman thought there were a large number of women who would prefer to have nothing to do with anything that looked like charity, and liked to be unhampered in their choice of a midwife. She strongly advocated the training of local women.

Dr. Boxall suggested that all applications for certificates should come through the local associations, to whom testimonials from clergy and doctors should also be sent.

Touching next upon the training, Mrs. Heywood Johnstone said that the cottage women were the most suitable class, and it was found that an ordinary fifth-standard education was quite sufficient. The best age was from 24 to 30. The limit for training in the Act was three months, but they would prefer six months.

Dr. Boxall said that there was a general consensus of opinion in favour of the cottage class of midwives, and thought that the midwives required in the rural districts should be dwellers in those districts, who would go and be trained and return to their own parts.

Dr. Griffith (Queen Charlotte's Hospital) thought that there would in future be two classes of midwives—one highly trained for the towns and the lesser trained for the country districts.

Dr. Cassidy, from the Royal Derbyshire Nursing Institution, gave some interesting details as to the work carried on by that institution.

Upon the subject of supervision there was a slight difference of opinion, Mrs. Heywood Johnstone and Dr. Holman speaking in favour of it, while Mrs. Rickman was opposed to it, and Dr. Percy Boulton feared that the midwives might supervise the committee.

Lord Belper being obliged to leave early, Mr. Heywood Johnstone, M.P., took the chair, and having summed up the main points of the discussion, the following resolutions were passed:—

(*a*) That the requirements of the Midwives Act may be usefully met by adding midwives to the staff of the local nursing associations, and by training local women to work under a small committee, provided that in both cases their work is kept distinct from cases dangerous to their calling.

(*b*) That where localities are unable to meet the initial expense of training, recourse must be had to grants from county councils and others, but some payment by the patient should always be made to meet the current expenses.

(*c*) That respectable women of the cottage class, carefully trained and working under supervision, will meet the requirements of the Act in rural districts.

(*d*) That the supervision of a local committee is advisable in every case, who should be in communication with the local supervising authorities under the Act.

A vote of thanks to Mr. and Mrs. Heywood Johnstone was then proposed by Sir Michael Foster, M.P., and the proceedings terminated.

### Appointments.

[No charge is made for announcements under this head, and we are always glad to receive, and publish, appointments. The information to insure accuracy should be sent from the nurses themselves, and we cannot undertake to correct official announcements which may happen to be inaccurate. It is essential that in all cases the school of training should be given.]

CLAYTON HOSPITAL, WAKEFIELD.—Miss Rosamund Dawson has been appointed sister. She was trained at Liverpool Royal Infirmary.

GUILDFORD UNION INFIRMARY.—Miss Julia Cope has been appointed assistant nurse. She was trained for three years at St. Peter's Home, Kelting, by the Meath Workhouse Nursing Association.

ST. LUKE'S HOSPITAL, HALIFAX.—Miss Eva Jones has been appointed night superintendent. She was trained at the Mill Road Infirmary and the Ladies' Charity or Lying-in Hospital, Liverpool. She has since been sister at the latter institution. She holds the L.O.S. certificate.

STOCKPORT INFIRMARY.—Miss Jessie J. Shaw has been appointed sister of children's wards. She was trained at West Ham Hospital, where she was afterwards staff nurse of the out-patients' department. She has since been staff nurse at the Children's Hospital, Pendlebury.

### Everybody's Opinion.

[Correspondence on all subjects is invited, but we cannot in any way be responsible for the opinions expressed by our correspondents. No communication can be entertained if the name and address of the correspondent are not given as a guarantee of good faith, but not necessarily for publication. All correspondents should write on one side of the paper only.]

### THE STATE REGISTRATION OF NURSES.

MRS. BEDFORD FENWICK writes: I must request you to correct an error in your report of the meeting held by the Royal British Nurses' Association on January 8th. You state that "Mrs. Bedford Fenwick also criticised unfavourably the appropriation of nurses' fees to the general expenses of the Board." I did no such thing. Manifestly the reason we nurses must pay a fee for registration is that the expenses of the organisation which conducts our business must be paid. I asked for information on a paragraph in Section 4 in the synopsis of the proposed Bill dealing with finance, to which the Chairman returned a satisfactory reply.

[Our representative states that the report is absolutely correct. Mrs. Bedford Fenwick, as our representative understood her, distinctly objected at the outset to the fees of nurses being devoted to the general expenses of the Board.—THE EDITOR, THE HOSPITAL.]

### DISTRICT NURSES AND PATIENTS' PAYMENTS.

"LANCASHIRE DISTRICT NURSE" writes: I should like to tell ex-Queen's Nurse that a District Nursing Association was started in Withwell, near Chorley, Lancashire, six months ago. Patients who wish to join the Association have to pay 3s. 6d. per annum, or if they call in the nurse without previously joining, the fee is 5s. This plan seems to be working very well and we hope that in eighteen months' time it will be self-supporting. I have four small villages to work, and there are two women on the committee in each village who collect the subscriptions either quarterly, half-yearly, or annually, as the patients prefer. In cases of great poverty the ladies of the executive committee pay part or the whole of their fees for them.

### ASSISTANT-NURSES IN WORKHOUSE INFIRMARIES.

MISS ANNIE LOUISA LEE writes: We must all agree with the statement made by a Poor-law matron in her letter to THE HOSPITAL that "in at least some of the largest Poor-law infirmaries the days of the assistant-nurse are past.' But I would ask, how is the nursing of the sick poor to be done in provincial workhouse infirmaries without the assistant-nurse? In these infirmaries, which are usually in the same building as the workhouse, the Guardians are often willing to appoint one fully-qualified nurse, but the nursing under her has to be done by assistant-nurses. Fully-qualified nurses will not accept these subordinate positions, nor will the Guardians offer sufficient salary to attract them. The salaries for these appointments vary from £20 to £30. I would ask, how are these appointments to be filled if assistant-nurses are not forthcoming to fill them?

### SIR WILLIAM BENNETT AND PRIVATE NURSING.

"A DUBLIN NURSE" writes: We, on this Irish side of the Channel, have been much interested by the perusal of Sir William Bennett's address on "Modern Nursing in Private Practice," and also in your comments thereon. From interchange of opinion in our Irish nursing world, of which I form a member, the address is regarded as being in the main an excellent one, but we do regret that you have not included in your own comments some pronouncement upon the crucial issue dealt with by Sir William under the heads of "The Refinement of the Modern Nurse" and "The Relation of Nurse to Patient." Irish nurses altogether take absolute exception to Sir William's views when he states that in the relation of nurse to patient in sickness "there is no such thing as 'decency or indecency.'" We Irish nurses have a full and grateful knowledge that the very contrary spirit actuates the medical and surgical professions in Ireland generally, and we should be sorry to think that it were otherwise with the professions in England or with our sister nurses in St. George's Hospital who listened to Sir William's words. In Dublin our hospital and nursing matrons are studious in joining, as is their duty, to protect their nurses from unsexing themselves by a detailed attendance on certain cases. Sir William appears to have addressed himself specially to the abandonment of "decency" as necessary in private nursing, in which, he says, a nurse will have to undertake any duties which are set down to her by the surgeon by whom she is employed. Now, there is surely no gradation of decency as between a hospital nurse and a private nurse? Then, it is not, at least in Ireland, once in a dozen cases that a nurse is "employed" directly by a medical man. Her services are requisitioned by the patient's family or friends, and most generally through a hospital or nursing home, whose matrons are, as I mention, studious to protect their nurses from association with indecency in regard to cases, no matter whether inside or outside of the wards. In your own words, we want "womanly women" as nurses; but not for cases of the nature indicated, which can only be decently ministered to in the manner of the hospital alluded to by Sir William Bennett, where care is taken that the nursing of cases of this class is conducted to a large extent by male attendants, in order to relieve the ordinary nurse from the necessity of dealing with them.

### MIDWIVES AND DOCTORS.

"COUNTRY MIDWIFE, L.O.S." writes: On entering my present post of midwife and parish nurse in a primitive parish, I was cordially greeted by the various doctors around who were pleased to have a certified person to relieve them of normal, and assist them in abnormal, cases amongst the very poor, of which this scattered parish is largely composed, and who, however willing, are unable to pay a doctor's fee. I am upwards of four miles from the nearest doctor, and the possible earliest assistance cannot generally be obtained in less than an hour or so. Most of the cottages are in very isolated positions, considerably off the high road, approached by bye-lanes across commons, so that the greater part of the journey must be traversed on foot. As I receive a stated salary our funds lose the special fee of 5s. on calling in a doctor, the nursing fee of 1s. 6d. per 10 days only being received. The doctor obtains his guinea for his visit at delivery with an understanding that he will be called, if his services are further required. The number of abnormal cases requiring chloroform in this district, compared with the town practice in which I was previously engaged, is surprising. It was my experience that doctors in town were not so ready to come to my assistance, though they were not sent for unless actually required, and could then leave their patients as soon as the case was over, feeling that they were in safe hands. And again, in ordinary sickness in these very rural places one can do much to save the doctor's time and the patients pain until his arrival, especially in cases of accident.

### REFORM IN HOSPITAL MANAGEMENT.

"H." writes: In a recent edition of your paper it was mentioned that there is always a danger in hospital management of assimilating the rules of the institution to the law of the Medes and Persians, and that now and again it is necessary to emphasise the fact that alterations are not only possible, but might be most desirable. It may be said that at all the London hospitals, and I have no doubt at most of the large provincial ones, there are regulations in force which prohibit the nurses from speaking to any of the students except on professional matters under severe penalties, whether those students be house-men, dressers, or what not, and whether they be inside or outside the hospital. So strictly is this rule enforced that even when a nurse is speaking to a student on professional matters she feels it is better for her own sake, should the matron enter, to stop talking than to run the risk of having to answer the charge of "talking to a student on unprofessional matters" in the matron's room on the following morning. Why should this be so? Why should not nurses be allowed to talk to students? The existing state of affairs tends to prevent the nurses and students having any communication whatever, whether on the subject of professional matters or not, a condition which is not only detrimental to the interests of these two parties, but also to those of the patients. Supposing, for the sake of argument, that there are grounds for maintaining the present relations inside the hospital, yet those grounds do not suffice to explain why the same relations should be maintained outside the hospital. Surely outside the hospital a nurse should have just as much freedom as any other woman; she has done with the hospital for the time being and should not be tied down by its regulations. To me it seems that the life of a probationer does not contain in it as much liberty as that of a schoolgirl, although she has, as a rule, passed the schoolgirl age years before. Let the regulations be such, then, that when a nurse throws off her uniform and adopts ordinary dress, she throws off at the same time the state of being a nurse and becomes an unfettered woman."

### The Nurses' Bookshelf.

"THE ART OF LAUNDRY WORK: PRACTICALLY DEMONSTRATED FOR USE IN HOMES AND SCHOOLS." By FLORENCE B. JACK, author of "Cooking for Invalids." Late Principal of the School of the Domestic Arts, Edinburgh. Sixth edition, revised and enlarged. (London: T. C. and E. C. J. Jack, 34 Henrietta Street, W.C., and Edinburgh. 1904. Price 2s. net.)

MISS JACK's book on laundry work shows that the author thoroughly knows her subject and can communicate her knowledge to others. From the preliminary sorting and soaking—or as Miss Jack, being a Scotswoman, calls it "steeping"—to the last touch of folding the washed and ironed garment, every step is made abundantly clear; and though the best methods are set forth and their superiority explained, those more hasty ways of doing things, to which people are occasionally forced by haste or lack of accommodation are not ignored, as they are in some textbooks. Miss Jack knows the failures at one point or other of the process of laundrying which so often befall the inexperienced worker, and explains how these can be prevented. The illustrations are all clear, and those which show how garments should be folded will be of the greatest value to the amateur who aspires to have the things she does look as well as those which are sent to a laundry. The book includes, besides instructions as to the washing and doing up of all ordinary articles, the treatment of stains of various kinds; the whitening, by a sulphur bath, of woollens that have become yellow; the washing of shawls, the comfort as well as the appearance of which is often lost after a single washing; the treatment of eider-down quilts, of black lace, of coloured embroidery, and other things which people more often like to wash at home than ordinary personal and household linen.

THE ZENANA, OR WOMAN'S WORK IN INDIA. Vol. X. (Published by S. Partridge and Co. Price 2s. 6d.)

THE bound volume of the monthly issues of the Zenana from November 1902 to November 1903 contains interesting information concerning zenana work, and will form an attractive gift-book for Christmas. The paragraphs on "Home News" and "Notes of the Month" practically constitute a diary of zenana mission events during the past year. We notice especially the endeavours to interest the young in this particular work, and so to insure its continuity, by the formation of societies, such as the Girl's Zenana Society, and also by means of special monthly "pages for the young" and examinations in mission subjects.

### Where to Go.

TENTH annual concert in aid of the Children's Hospital Hackney Road, in Shoreditch Town Hall, 7.45 P.M. on February 4th.

### Echoes from the Outside World.

### Royal Visit to the Gordon Boys' Home.

ON Monday afternoon the King and Queen paid a private visit to the Gordon Boys' Home at Woking. They were received by a number of members of the Committee, and the King then made a most minute inspection of the lads, who presented a most soldierly appearance, receiving his Majesty with a Royal salute and afterwards marching past in company column to the strains of their band. A visit was next paid to the chapel, to the erection of which their Majesties had contributed, and the memorial tablet to the late Duke of Clarence was inspected. In the chapel the choir of boys, under the direction of the chaplain, sang an anthem and a carol, the organ being played by a boy of 14 named Winter. The Queen asked that Winter might be presented to her. Both the King and Queen spoke to several of the boys on their round of instruction, and in the gymnasium the Queen singled out a little lad named Dibble, who although much the smallest boy in the Home, is one of the best gymnasts. One of the instructors was wearing the King's war ribbon without the medal, and his Majesty, noticing this, asked Sir George Higginson to see that the medal was applied for. Visits were also paid to the workshops, the dining-hall, and the hospital, which is almost empty, testifying to the excellent health of the boys. Finally, the King gave a short address to the boys in the recreation-hall. He said that he was pleased to see them looking so well and happy and learning so much. When they went out into the world he hoped that they would show that they had benefited by their training, whatever occupations they adopted. He trusted that above all they would remember him in whose honour the Home was founded.

### The New Governor of the Straits Settlements.

THE King has approved of the appointment of Sir John Anderson, K.C.M.G., to be Governor and Commander-in-Chief of the Straits Settlements and High Commissioner of the Federated Malay States in the place of Sir Frank A. Swetenham. Sir John Anderson has risen entirely by merit. The son of the superintendent of the Gordon Mission at Aberdeen, he had a brilliant career at the Aberdeen University, entered the Colonial Office as second-class clerk in 1879, and has steadily worked his way up. He was attached to the staff of the Behring Sea Arbitration in London and Paris, was secretary to the conference between Mr. Chamberlain and the Colonial Premiers, and attended the Prince of Wales on his visit to the Colonies as Colonial Office representative. In 1901 he received the K.C.M.G. in recognition of his services, and he now, amid general congratulations, assumes one of the most important of Colonial Governorships.

### Release of Arthur Lynch.

IT will be recollected that on January 23rd, 1903, Arthur Lynch was found guilty of high treason in the High Court of Justice for his proceedings in South Africa and sentenced to death by Mr. Justice Wills. The sentence was almost immediately commuted to penal servitude for life. Exactly a year later, on Saturday last, Lynch was released by license from Lewes Gaol by order of the Home Secretary. He has, therefore, while regaining his liberty, been put upon his good behaviour. Moreover, being a convict at large on license, he cannot sit in Parliament. It was not supposed that he would remain in prison for a prolonged period.

### Destruction of a Norwegian Town.

A GREAT calamity has occurred in Norway. In the early hours of Saturday morning last the town of Aalesund on the west coast, was practically wiped out of existence by fire. Only two lives were lost, but upwards of 10,000 people have been rendered homeless, and enormous damage has, of course, been done to property. The cause of the conflagration has not yet been ascertained. From first to last however, it swept everything before it, from fine stone buildings, including a handsome church and school, to cottages. Thousands of victims had to spend 24 hours in the open fields in a storm of cold wind and rain, without food. The trade of Aalesund, which is, or was, spread over two small islets, is chiefly derived from fishing, and has lately increased at a great rate. At the suggestion of the German Emperor, a relief committee has been organised at Berlin with the co-operation of the Red Cross Society, and on Sunday afternoon, the steamship *Phœnicia* left Hamburg for Aalesund with supplies for 4,000 persons. Three doctors and a staff of male and female nurses were on board. Subscription lists for the sufferers from the fire have been opened to which the King and Queen of Norway have contributed.

### Tragic Death of Mr. Whitaker Wright.

THE trial of Mr. Whitaker Wright, who was charged with making false statements in order to deceive and defraud shareholders in the London and Globe and certain other companies, closed on Tuesday with a startling tragedy. The judge, Mr. Justice Bigham, having summed up, the special jury retired at two o'clock to consider their verdict. At the end of one hour they returned into court with a verdict of guilty on all counts of the indictment. Mr. Lawson Walton, K.C., who defended the prisoner, then addressed the judge in mitigation of sentence, but his lordship said that he could not conceive a worse case and inflicted the severest sentence possible, namely, seven years' penal servitude. The prisoner, who protested his innocence, was removed in custody, but immediately became seriously ill, and an hour after the sentence was pronounced he expired.

### Music.

THE concert given by Miss Marie Hall on Friday at St James's Hall was patronised by an enthusiastic crowd, and there are no signs of any falling off in interest with regard to the artistic performances of the young performer. Miss Hall completed the arduous task she had set herself of playing the solo portions of three concertos—two of these ranking as master works—with evident delight to her audience and much credit to herself. Her tone is far more powerful than it was, and her playing of Mendelssohn's Concerto was exquisitely delicate, but perhaps Tchaikovsky's Concerto was the most successful of the three. Here she repeated the triumph of her first appearance.

### Fog and Frost.

AT the end of last week London was visited in places with a very thick fog, so dense on Friday, about Blackfriars, that it was as if a black pall lay over the City, and everywhere artificial light had to be resorted to. In Hyde Park many persons lost their way, and torch boys reaped a good harvest There were collisions at Clapham Junction and Peckham Rye on Saturday, the former without much hurt to anyone but the guard, the latter causing rather serious injury to two ladies. An omnibus was overturned owing to the fog, and the occupants taken to the hospital, several cases of drowning occurred, and at Hammersmith a well-known juggler walked off the platform of the District Railway on to the line, falling headlong. In Canada the temperature in places is 48 degrees below zero. New York has been visited by a heavy fog, and Long Island Sound is a compact ice-field from shore to shore, from Hell Gate to Newhaven. The great floe is 60 miles long and 12 miles wide at Newhaven, and through the centre is a narrow strip of thin ice which marks the path broken by the big Fall River steamboats.

### Notes and Queries.

FOR REGULATIONS SEE PAGE 135.

*False Noses.*

(150) *Matron* would be glad to know of a good firm from which false noses may be obtained, and the probable cost.

Ask the surgeon in charge of the case, or apply to the Surgical Aid Society, Salisbury Square, Fleet Street, E.C.

*Age Limit.*

(151) Can you let me know what is the age limit for entrance to the examination in maternity nursing for the L.O.S. certificate?—*Nurse J.*

The London Obstetrical Society does not limit the age of candidates for its examination provided they are over 21.

*Sea-going Nurses.*

(152) To whom should I apply for information respecting the regulations regarding trained nurses on board ship?—*E. Q. N.*

Write to Miss Penn, The Cottage, New Shoreham, Sussex.

1. Where shall I obtain all particulars as to nurses engaged on first-class steam vessels? 2. Is one year's training in a cottage hospital of any value in obtaining a situation as nurse-matron in a boarding school?—*Nurse Lindsay.*

1. See reply to *E. Q. N.* 2. Of great value.

Kindly tell me how a trained nurse should apply for a position as stewardess on board one of the large steam-ships sailing to India, &c.—*M. J. L.*

See reply to *Nurse Lindsay* and apply to the Secretaries of the various companies. The supply greatly exceeds the demand.

*Salary.*

(153) This is a small hospital of nine beds; but in a few weeks we are to go to a new one containing ten beds and two private wards. Should I be justified in asking for an increase of salary?—*Miss H.*

If your duties are heavier, certainly.

*Hospital Training.*

(154) I have just finished two years' fever training, and during that time I have never seen any sort of operation performed, nor have I ever passed a catheter, etc. I have had plenty of physiology lectures and a few fever lectures. Could I conscientiously take a post as charge nurse in another fever hospital and attempt to train probationers?—*Cara.*

You could, of course, only undertake such duties as you have yourself learnt and teach them to others.

*Abroad.*

(155) I am going out to relatives in Hobart, Tasmania, but after paying them a short visit I should like to go on with my work. Could you kindly tell me of a hospital where I could apply?—*Nurse.*

The Matron, the General Hospital, Hobart, might help you.

*Naval Nursing.*

(156) 1. Will you kindly tell me to whom to apply forinformation respecting Queen Alexandra's Naval Nursing Service? 2. What good nurse's dictionary would you recommend?—*A. A. P.*

1. Apply to the Director-General, Medical Department of the Navy, Admiralty, Craven House, Northumberland Avenue, W.C. 2. Miss Honnor Morten's "Nurse's Dictionary of Medical Terms and Nursing Treatment" (Scientific Press) is very complete. Price 2s.

*Registration. Fœtal Skull.*

(157) 1. Will you kindly tell me where to apply to be registered as a midwife? 2. and where I could borrow a pelvis and fœtal head for a week?—*A. R.*

1. Apply to the Secretary, the Central Midwives Board, 6 Suffolk Street, Pall Mall, S.W. 2. Write to the Midwives Institute, 12 Buckingham Street, Strand, W.C.

### Standard Nursing Manuals.

"The Nursing Profession: How and Where to Train." 2s. net; 2s. 4d. post free.

"Nursing: Its Theory and Practice." (Revised Edition). 3s. 6d. post free.

"Elementary Anatomy and Surgery for Nurses." By William McAdam Eccles, M.D.Lond., M.B. 2s. 6d. post free.

"Elementary Physiology for Nurses." By C. F. Marshall, M.D., B.Sc., F.R.C.S. 2s. post free.

"Ophthalmic Nursing." By Sydney Stephenson, M.B., F.R.C.S., 3s. 6d. net; 3s. 10d. post free.

"Nursing in Diseases of the Throat, Nose, and Ear." By P. Macleod Yearsley, F.R.C.S.Eng., M.R.C.S. 2s. 6d. post free.

"Fevers and Infectious Diseases." 1s. post free.

### For Reading to the Sick.

A PRAYER.

I ASK Thee for the daily strength
To none that ask denied,
And a mind to blend with outward life,
While keeping at Thy side;
Content to fill a little space,
If Thou be glorified.

And if some things I do not ask
In my cup of blessing be,
I would have my spirit filled the more
With grateful love to Thee;
And careful, not to serve Thee much,
But to please Thee perfectly.

Blest be Thy love, dear Lord,
That taught us this sweet way,
Only to love Thee for Thyself,
And for that love obey.

*J. Austin.*

If ever human love was tender, and self-sacrificing, and devoted; if ever it could bear and forbear; if ever it could suffer gladly for its loved ones; if ever it was willing to pour itself out in a lavish abandonment for the comfort or pleasure of its objects; then infinitely more is Divine love tender, and self-sacrificing, and devoted, and glad to bear and forbear, and to suffer, and to lavish its best of gifts and blessings upon the objects of its love. Put together all the tenderest love you know of, the deepest you have ever felt, and the strongest that has ever been poured out upon you, and heap upon it all the love of all the loving human hearts in the world, and then multiply it by infinity, and you will begin, perhaps, to have some faint glimpse of what the love of God is.—*H. W. S.*

All we have to do is so to live that we do not hinder the light of Christ within from "its full and perfect work." What would Jesus do? What would Jesus say? How would Jesus bear this trial? Such secret thoughts as these will help us too to live a Christlike life, and the strong, pervading force of the Holy Spirit within us will do the Master's work, and glorify the Father even through us, if we but keep our thoughts, our love, our wills so centred on Him that He can shine out from the temple of our souls.

*E. A. D.*

Faithful unto death. We often think of it as if it must demand a long as well as an unflinching effort: and so indeed it may demand, but we know not whether it will. One moment may suffice, for aught we truly know to the contrary. One moment's effort: the weakest might undertake so much. Or, if ever so long a strain be required, God's strength is always stronger than strong enough.

*Christina Rossetti.*

For His great love has compassed
Our nature, and our need
We know not; but He knoweth,
And He will bless indeed.
Therefore, O heavenly Father,
Give what is best to me;
And take the wants unanswered,
As offerings made to Thee.

*Anon.*

